# Genome Sequences for Extended-Spectrum Beta-Lactamase-Producing Escherichia coli Strains Isolated from Different Water Sources

**DOI:** 10.1128/MRA.00328-21

**Published:** 2021-06-17

**Authors:** Holly A. Gray, Patrick J. Biggs, Anne C. Midwinter, Sara A. Burgess

**Affiliations:** a *^m^*EpiLab, School of Veterinary Science, Massey University, Palmerston North, New Zealand; b School of Fundamental Sciences, Massey University, Palmerston North, New Zealand; University of Maryland School of Medicine

## Abstract

Extended-spectrum beta-lactamase (ESBL)-producing *Enterobacteriaceae* are considered a critical priority by the World Health Organization. Presented here are two genome sequences of Escherichia coli strains isolated from New Zealand freshwater. The genome sequences’ mean size was 5.2 Mb, with a mean of 4,848 coding sequences. Both genomes carried the ESBL *bla*_CTX-M_ gene.

## ANNOUNCEMENT

New Zealand faces increasing rates of extended-spectrum beta-lactamase-producing *Enterobacteriaceae* (ESBL-E) found in the community from urinary sources (1). Escherichia coli is the predominant species of ESBL-E detected in human clinical specimens, increasing from 50.2% in 2007 to 74.1% in 2016 in New Zealand (2, 3). However, ESBL-producing E. coli organisms are also being detected in wastewater and the wider environment, which could contribute to the increasing rates of resistance spread (4, 5).

Long-read Oxford Nanopore Technologies (ONT) sequencing was performed on two E. coli isolates originating from the Manawatū River and a stormwater drain which feeds into the river, 18.1 km apart. The samples were processed by filtering 100  ml of water using mixed cellulose ester filters (0.45 μm; Millipore, Germany) and enrichment in 10  ml of buffered peptone water (BD Difco, Becton, Dickinson, USA) overnight at 35°C. Each enrichment was subcultured on ESBL chromogenic agar (CHROMagar; Fort Richard Laboratories, Auckland, New Zealand) and incubated at 35°C overnight, and a single colony was streaked onto Columbia horse blood agar (Fort Richard Laboratories). A single colony was inoculated into 5 ml of lysogeny broth and incubated overnight at 35°C for DNA extraction. The Promega Wizard high-molecular-weight kit (Madison, WI) was used for DNA extraction, following the kit instructions, omitting the proteinase K. The DNA quantity and quality were checked using fluorescent spectrometry (Qubit 2.0; Invitrogen, USA) and gel electrophoresis, but the DNA was not size selected or sheared. Long-read ONT sequencing libraries were prepared using the rapid barcoding kit (SQK-RBK004). The isolates were sequenced using a MinION Mk1B device (Oxford Nanopore Technologies, Oxford, England) and a SpotON flow cell R9 version FLO-MIN106D.

Default parameters were used for all software, unless stated. Base calling was conducted using Guppy v.4.2.2 (https://github.com/nanoporetech/pyguppyclient), and reads were demultiplexed using qcat v.1.1.0 (https://github.com/nanoporetech/qcat) (6). Adapters were removed using Porechop v.0.2.4 (https://github.com/rrwick/Porechop) and reads filtered using Filtlong v.0.2.0 (https://github.com/rrwick/Filtlong), keeping 95% of the reads with a minimum length of 1,000 bp (7, 8). The read quality was assessed using NanoStat v.1.5.0 (9). Hybrid assembly was achieved with Unicycler v.0.4.9 (10). The assemblies were visualized using Bandage v. 0.8.1 (11), and the assembly quality was assessed using Quast v.5.0.2 (https://github.com/ablab/quast) (12). The sequence type, plasmid type, and resistance genes were identified using MLST v.2.0, PlasmidFinder v.2.0, and ResFinder v.4.1, respectively (13–15). Annotation of the assembled genome utilized the NCBI Prokaryotic Genome Annotation Pipeline v.5.1 (16). The sequencing, assembly, and genome details are presented in Table 1.

**TABLE 1:** Strain characteristics and genomic statistics

Attribute	Information for isolate:	
	SB0258h1	SB0283h1
Sample date	15 July 2019	17 November 2019
Sample source	Manawatū River, Tipp Road, Palmerston North	Centennial Drive storm water drain, Palmerston North
GPS coordinates for sample source site	–40.387886, 175.582538	–40.373294, 175.626352
Species	E. coli	E. coli
Total no. of ONT bases post-adapter removal	506,915,502	831,620,531
No. of ONT reads	143,626	187,713
*N*_50_ (bp) after adapter removal	6,735	8,322
*N*_50_ (bp) after filtering	7,140	13,347
Depth (×)	97	160
Assembled genome length (bp)	5,227,361	5,207,710
No. of contigs	1	9
No. of CDS	4,884	4,812
Complete circular chromosome?	Yes	Yes
GC content (%)	50.7	50.63
Sequence type	ST38^*a*^	ST1722
Plasmid types	ND^*b*^	IncFIA, IncFIB, IncFII
ESBL/AmpC gene type	*bla*_CTX-M-24_	*bla*_CTX-M-15_
Other beta-lactamase genes	ND^*b*^	*bla*_TEM-1B_, *bla*_OXA-1_
Other genes coding for resistance	*sul1*, *dfrA17*, *aac(3)-IId*, *aadA5*, *mph*(A)	*catB3*, *mph*(A), *mdf*(A)
GenBank accession no.	CP071954	JAGEVC000000000
SRA accession no.	SRR13999307	SRR13999306
BioSample accession no.	SAMN18350774	SAMN18352327

a ST, sequence type.

b ND, not detected.

The mean assembled E. coli genome size was 5.2 Mb, with a mean GC content of 50.67% and a mean of 4,848 coding sequences. The assembled SB0258h1 genome was complete and contained a chromosomal ESBL gene, *bla*_CTX-M-24_. The SB0283h1 assembly consisted of one complete circular chromosome and eight linear contigs, one of which was a partial plasmid containing the ESBL gene *bla*_CTX-M-15._ This study demonstrates the need for further measures to reduce the spread of antibiotic resistance.

### Data availability.

The draft genome assemblies have been deposited in GenBank under BioProject accession number PRJNA715472.
